# Employing diclofenac sodium as a novel therapeutic frontier for *Staphylococcus epidermidis* infections

**DOI:** 10.1038/s41598-025-14316-1

**Published:** 2025-08-26

**Authors:** Amal M. Abo-Kamar, Ahmed A. Abdelaziz, Alaa E. Ashour, Moataz A. Shaldam, Engy Elekhnawy

**Affiliations:** 1 Microbiology and Immunology Department, Faculty of Pharmacy, Tanta University, Tanta, 31527 Egypt; 2 Department of Pharmaceutical Chemistry, Faculty of Pharmacy, Kafrelsheikh University, Kafr El-Sheikh, 33516 Egypt

**Keywords:** Systemic infection, Molecular docking, QRT-PCR, Histopathology, Biofilm, Microbiology, Medical research

## Abstract

The spread of biofilm-forming multidrug-resistant pathogenic bacteria is an alarming public health issue requiring significant research. Drug repurposing is a novel approach to combating bacterial infections that is currently being studied. Here, we explored diclofenac sodium’s potential antibacterial and antibiofilm action on *Staphylococcus epidermidis* bacteria. Diclofenac sodium revealed antibacterial action on *S. epidermidis* isolates with minimum inhibitory concentrations of 500 to 2000 µg/mL. It also exposed antibiofilm action using the crystal violet assay and scanning electron microscope. Using qRT-PCR, diclofenac sodium has downregulated the expression of the biofilm genes (*cna*, *fnb*A, and *ica*) in 20% of the isolates. An animal model revealed the effect of diclofenac sodium on a systemic infection with *S. epidermidis* in mice. Diclofenac sodium has improved the liver, spleen, and kidney architecture. Molecular docking was used to explore the possible mechanism for the activity of diclofenac sodium against *S. epidermidis*, which revealed the high affinity of diclofenac sodium toward *S. epidermidis* protein and TcaR enzymes. Thus, diclofenac sodium could be a clinical solution for disseminating resistance among *S. epidermidis* and could be investigated for its combination with different antibiotics in future studies.

## Introduction

*Staphylococcus epidermidis* is a Gram-positive bacterium and one of the opportunistic pathogens commonly encountered in immunocompromised patients^1^. It can trigger different ailments, ranging from mild skin infections to life-threatening illnesses, like endocarditis, osteoarticular infections, and medical device-related infections, which are primarily associated with biofilm formation^2–4^.

Biofilm comprises bacterial cells attached to the colonized surface^5–7^. Bacterial biofilms help bacteria evade the immune system’s response and can be less sensitive to antibiotic treatment^8–10^. As a result of the failure of antibiotics to treat biofilm-related infections owing to the augmented endurance of the cells and the inability to enter into the complex structure of biofilm, resistance to many antibiotics is more likely to occur than in their planktonic counterparts^11,12^. So, finding an alternative that can affect biofilms is necessary, either by stopping their formation or disrupting them^13,14^.

Overuse of antibiotics promotes the occurrence of multidrug-resistant (MDR) pathogens^15^. Antimicrobial resistance lengthens hospitalization, increases healthcare costs, and increases morbidity and mortality rates. *S. epidermidis* and other Gram-positive pathogenic bacteria have become a main healthcare problem because of the fast resistance rate to the commonly used antibiotics^16,17^. Consequently, novel tactics are mandatory to combat such pathogens, such as repurposing FDA-approved drugs to manage the infections caused by these bacteria. Drug repurposing, repositioning, or re-tasking is a tactic for recognizing novel uses for FDA-approved drugs outside their original scope of medical indication. This approach has gained significant attention as discovering new antibiotics is a lengthy and costly procedure with a relatively low rate of success^18,19^.

Here, we aimed to expose the potential antibacterial action of diclofenac sodium against *S. epidermidis* clinical isolates using different techniques, including in vitro, in vivo employing an animal model, and in silico using molecular docking analysis.

## Materials and methods

### Bacteria

*S. epidermidis* (*n* = 20) were from the blood, wound, sputum, and ear of Mansoura University Hospital patients, Egypt. The samples were taken from the patients for routine diagnosis, specifically for this study. The patient data were obscured. The bacterial colonies were identified by Gram-staining and biochemical identification, as previously described^20^.

### Antibiotic sensitivity test

Kirby-Bauer disk diffusion was employed to test the antibiotic sensitivity of *S. epidermidis*^21^ on Mueller-Hinton agar (MHA) plates. The subsequent antimicrobials were tested, oxacillin (OX; 1 µg), azithromycin (AZM; 15 µg), erythromycin (E; 15 µg), chloramphenicol (C; 30 µg), gentamicin (GN; 10 ug), trimethoprim-sulfamethoxazole (SXT; 1.25/23.75 µg), linezolid (LZD; 30 µg), clindamycin (DA; 2 µg), tetracycline (TE; 30 µg), minocycline (MI; 30 µg), gatifloxacin (GAT; 5 µg), and ciprofloxacin (CIP; 5 µg). In brief, after spreading the bacterial suspension on the surface of the MHA agar, the antibiotic discs were applied onto the surface and the plates were incubated at 37℃ for 24 h. Then, the inhibition zones were measured and interpreted.

### Antibacterial potential of diclofenac sodium

It was performed using agar well diffusion assay as previously described^22–25^. On the surface of the MHA plates, about 100 µL of each tested microorganism (0.5 McFarland) was distributed evenly, and each well received diclofenac sodium (4000 µg/mL). The antibacterial activity was inspected by forming inhibition zones around each well after the plates were incubated at 37 °C for 24 h, and sterile water was utilized as a negative control.

### Minimum inhibitory concentration of diclofenac sodium (MIC)

Broth microdilution was utilized in MH broth to detect MIC values of diclofenac sodium against MDR *S. epidermidis* isolates. After overnight incubation of the tested drug with the tested isolates in microtiter plates at 37 °C, the growth of the tested bacteria in the wells of the plates was detected according to the broth turbidity. By visual inspection, the lowest diclofenac sodium concentration that showed no growth was recorded as MIC^26–28^.

### Biofilm Inhibition assay

The semi-quantitative biofilm formation assay was employed in the current study^29–31^ with some modifications before and after treatment with diclofenac sodium. Bacterial suspensions in tryptone soy broth (TSB) were prepared from an overnight culture and were adjusted to 10^6^ CFU/mL in freshly prepared culture broth of TSB. Then, 200 µL of the bacterial suspension was transferred into the wells of microtiter plates in the presence and absence of sub-MIC of diclofenac sodium, and they were incubated at 37 °C for 48 h. Then, TSB was gently removed, and the plates were washed with distilled water to remove any planktonic cells, with subsequent air drying. After 20 min of treatment with 200 µL of 99% methanol for fixation, the formed biofilms were stained with 200 µL of 1% crystal violet (CV) solution for 15 min. After washing the plate, 33% glacial acetic acid was used as a solvent for CV, and the absorbance of the solubilized dye was measured at 570 nm with a spectrophotometer. The effect of diclofenac sodium was tested by incubating the tested bacteria with 0.5 MIC.

### Scanning electron microscope (SEM)

As previously described, the antibiofilm action of diclofenac sodium on *S. epidermidis* isolates was visualized using SEM (JEOL, Japan)^32^.

### Relative gene expression

The effect of diclofenac sodium on the biofilm genes in *S. epidermidis* (*cna*, *fnb*A, and *ica*) was elucidated by qRT-PCR^33,34^. The isolates were grown in tryptone soy broth (TSB) in the presence and absence of 0.5 MICs of diclofenac sodium and incubated overnight at 37 °C. After incubation, cells were harvested by centrifugation and immediately stored at − 80 °C. The RNA from *S. epidermidis* isolates was extracted and purified using TRIzol reagent (Life Technologies, USA) following the manufacturer’s protocol. Reverse transcription for cDNA synthesis was done using a reverse transcription kit (Qiagen, USA). After that, the cDNA was amplified using SYBR green master mix (Thermo Scientific, USA). The average threshold cycle (CT) values were normalized to the housekeeping gene (16 S rRNA) and its sequence is forward 5′GGGACCCGCACAAGCGGTGG3′and reverse 5′GGGTTGCGCTCGTTGCGGGA3′^35^. The seuences of *ica* gene is forward 5′GAGGTAAAGCCAACGCACTC3′ and reverse 5′CCTGTAACCGCACCAAGTTT3′. The sequence of *the fnb*A gene is forward 5′AAATTGGGAGCAGCATCAGT3′ and reverse 5′GCAGCTGAATTCCCATTTTC3′. The sequence of *the cna* gene is forward 5′AATAGAGGCGCCACGACCGT3′ and reverse 5′GTGCCTTCCCAAACCTTTTGAGC3′^36^. Only genes with ≥ two-fold changes (either increased or decreased) were regarded as statistically significant^37^.

### Systematic infection

Thirty male mice weighing 25–30 g and aged 6–8 weeks were grouped into five groups, each with six mice. The animals were purchased from the animal house of Cairo University. The first group was a normal control. The remaining four groups were infected with 0.1 mL via intravenous injection of 1.5 × 10^7^ colony-forming units (CFU) of *S. epidermidis*. The second group served as a positive control group, and the third group was administered linezolid (80 mg/kg/12 h) orally as a standard drug. The fourth and fifth groups administered diclofenac sodium orally with doses of 20 mg/kg/24 h and 40 mg/kg/24 h, respectively^38,39^. The experimental procedures were approved by the ethics committee at the faculty of pharmacy, Tanta University (TP/RE/3/24 p-01).

### Histopathological analysis

After two weeks, mice were euthanized by cervical dislocation and the animals were anaesthetized by isoflurane inhalation. Liver, spleen, and kidney samples were obtained from each group. Tissue samples (2 × 3 mm) were excised, fixed in buffered formalin (10%), and treated as previously described^40^, and finally stained with hematoxylin and eosin (H&E) and photographed using a light microscope.

### Molecular Docking

The crystal structures of the target proteins of *S. epidermidis* were obtained from the protein data bank (PDB) and the AlphaFold database. AutoDock Vina was used for the docking studies on diclofenac sodium^41^. Ligand structures were drawn into Marvin Sketch V22.2^42^, and the most energetically favored conformer was exported as a (*.pdb) file format. Water molecules were removed, and hydrogen-assigned gastier charges were performed using AutoDock tools. The centers and sizes of the grid boxes used to define the active site for each receptor are exposed in Table 1. The 3D visualization and 2D schematic presentation were generated by BIOVIA Discovery Studio Visualizer^43^.

**Table 1:** Target proteins in *S. epidermidis* and their PDB code and grid box information.

Protein	S. epidermidis protein	IcaC	TcaR
PDB	3KP3^44^	AF-A0A162GV30-F1^45^	3KP4^44^
Grid coordinates (x, y, z)	−40.3, 4.1, −3.9	4.5, −4.0, −6.1	−26.3, 31.4, 4.3
Grid Size (x, y, z)	15.6, 15.4, 20.7	20.5, 29.0, 39.2	14.5, 16.9, 19.9

### Statistics

ANOVA was employed to disclose the consequence among the examined groups using GraphPad Prism software (USA) at *p* < 0.05.

## Results

### Bacterial isolates and antibiotic sensitivity test

**Fig. 1:**
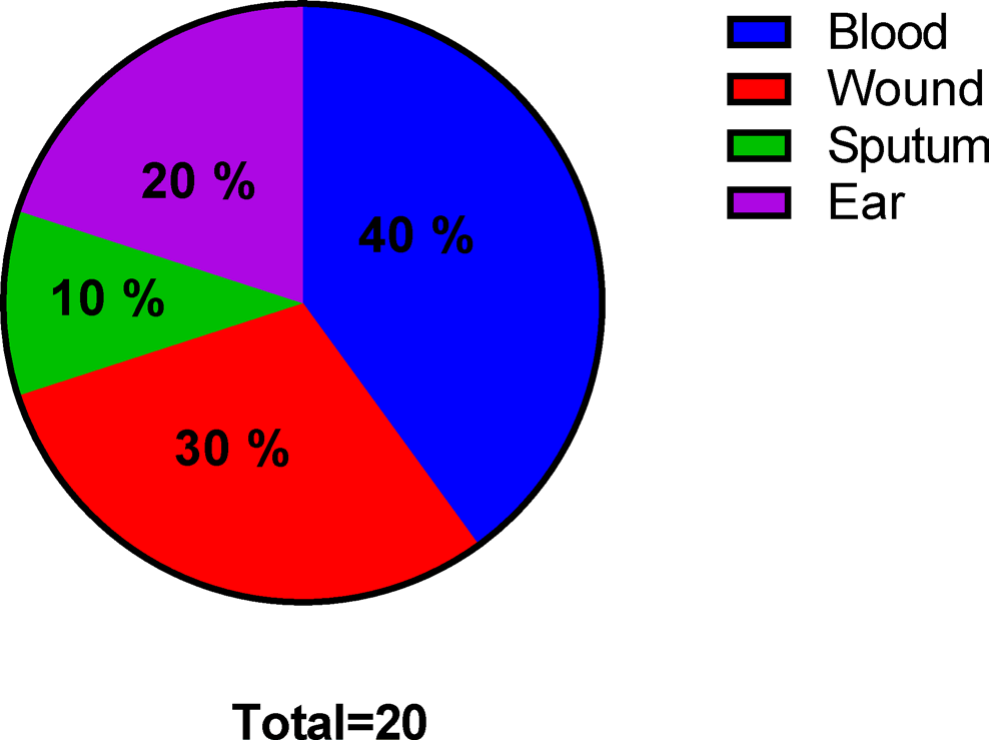
Pie graph revealing the specimens of *S. epidermidis* isolates.

*S. epidermidis* (*n* = 20) was isolated from diverse specimens, including blood (8), wound (6), sputum (2), and ear (4), as revealed in Fig. 1.

**Fig. 2:**
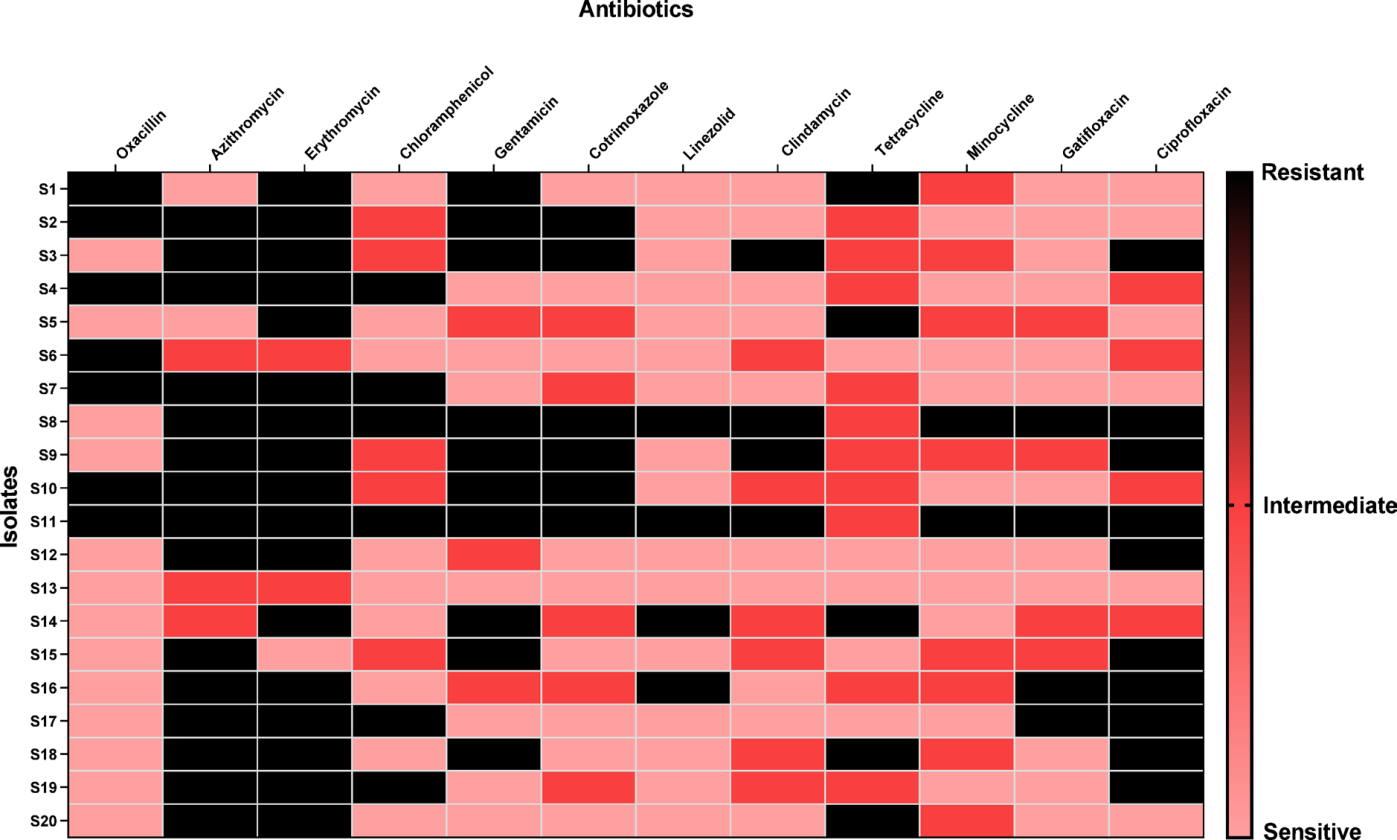
Heat map revealing the antibiotic susceptibility of the tested isolates.

The antibiotic resistance percentages were as follows: oxacillin (40%), azithromycin (75%), erythromycin (85%), chloramphenicol (30%), gentamicin (45%), trimethoprim-sulfamethoxazole or cotrimoxazole (30%), linezolid (20%), clindamycin (20%), tetracycline (25%), minocycline (10%), gatifloxacin (20%) and ciprofloxacin (50%). Figure 2 reveals the percentages of resistance among the tested isolates.

### MICs of diclofenac sodium

Broth microdilution was utilized to calculate the MICs of diclofenac sodium, which ranged from 500 to 2000 µg/mL.

### Antibiofilm action

Biofilm formation was estimated before and after treatment with diclofenac sodium at 0.5 MICs. Interestingly, diclofenac sodium repressed the biofilm formation in 45% of the tested isolates (Table 2).

**Table 2:** Impact of diclofenac sodium on the biofilm-forming ability of *S. epidermidis* isolates.

Isolate code	Biofilm formation ability*	
	Before treatment	After treatment
S2	SBF	NBF
S4	SBF	NBF
S6	MBF	NBF
S9	SBF	NBF
S13	MBF	NBF
S15	SBF	WBF
S16	MBF	WBF
S17	SBF	WBF
S20	MBF	WBF

* SBF means strong biofilm forming, MBF means moderate biofilm forming, WBF means weak biofilm forming, and NBF means non-biofilm forming.

### SEM

**Fig. 3:**
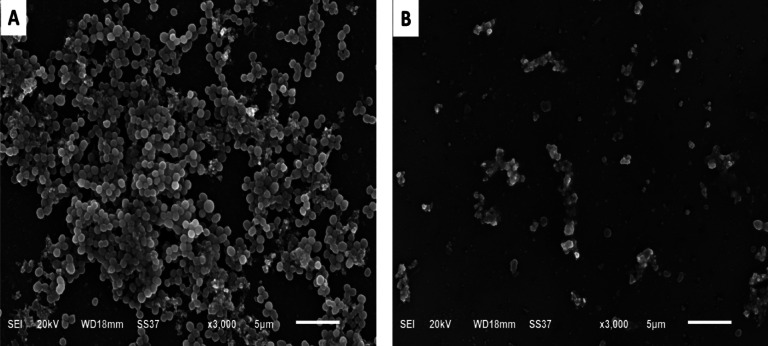
Impact of diclofenac sodium on biofilm formation using SEM, where (**A**) before and (**B**) after treatment.

The ability of diclofenac sodium to elicit antibiofilm action was additionally established by SEM. Results showed that the biofilm was disturbed by diclofenac sodium. The number of cells diminished in the treated biofilm-forming isolates in relation to the untreated ones (Fig. 3).

### qRT‑PCR

**Fig. 4:**
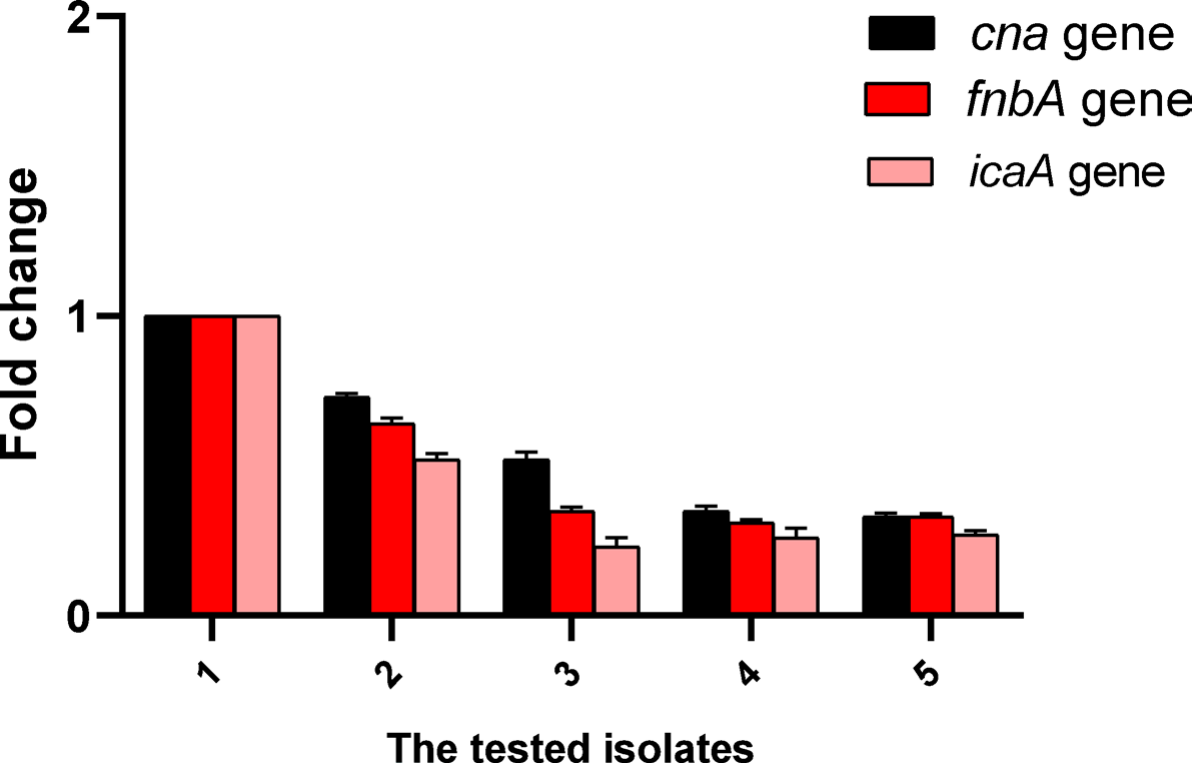
Fold change of the biofilm genes revealed by qRT-PCR after treatment with diclofenac sodium.

To study the phenotypic inhibition of virulence factors by diclofenac at the genetic level, qRT-PCR was employed to reveal the influence of diclofenac sodium on the expression of biofilm genes. Diclofenac sodium showed a significant down-regulating effect on the biofilm genes in 20% of the isolates (Fig. 4).

### Histopathology of liver, spleen, and kidney

**Fig. 5:**
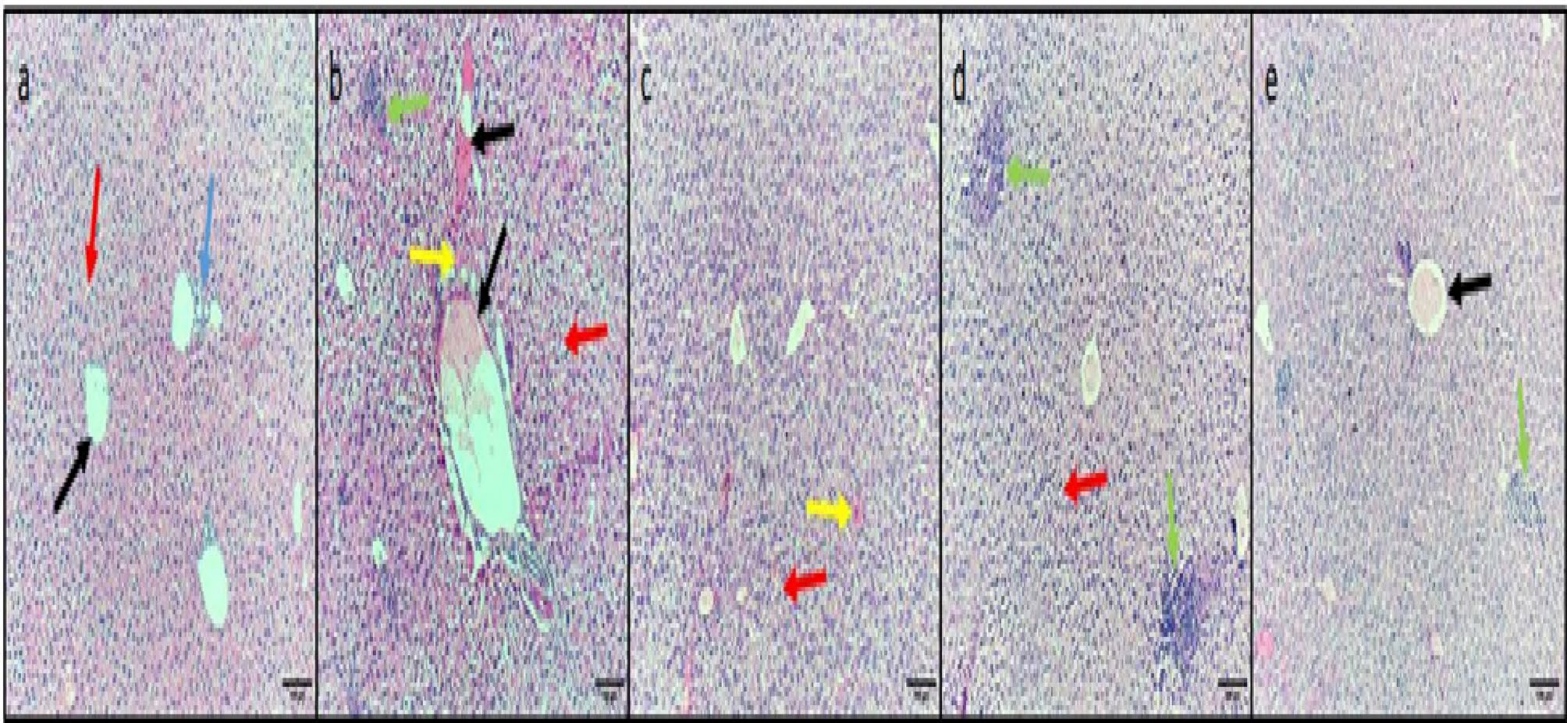
H&E micrographs of liver sections of (**a**) Normal control group displaying average-sized central veins (black arrow) and portal triad (blue arrow) bounded by cords of hepatocytes separated by sinusoids (red arrow). (**b**) The positive control group displayed congested vessels (black arrows) bounded by hepatocytes with hydropic degeneration (red arrow), inflammatory cells (green arrow), and apoptotic bodies (yellow arrows). (**c**) The standard drug-treated group displayed a restoration of normal-sized veins and no inflammation, but with mild hydropic degeneration (red arrow) and apoptotic bodies (yellow arrow) in the hepatocytes. (**d**) Diclofenac sodium group (20 mg/kg) displayed a mildly dilated-sized central vein surrounded by cords of hydropic degenerated hepatocytes (red arrows) and multiple inflammatory cell foci (green arrow). (**e**) Diclofenac sodium group (40 mg/kg) displayed a mildly dilated-sized congested central vein (black arrow) surrounded by cords of normal hepatocytes and inflammatory cell infiltration (green arrow)—100× magnification.

**Fig. 6:**
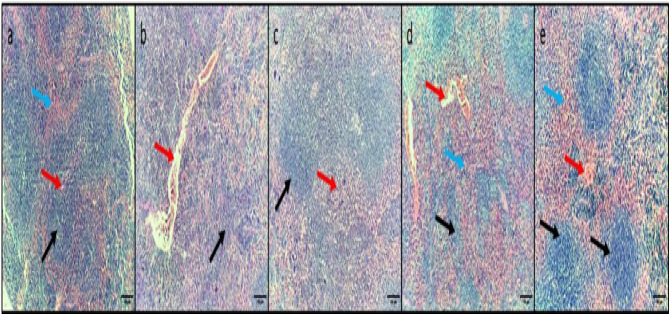
H&E micrographs of spleen sections of (**a**) Normal control group displaying normal-sized white pulp (lymphoid follicles) (black arrows) with central arteriole (red arrow) surrounded by average-sized red pulp (blue arrow). (**b**) The positive control group displayed marked congestion, exhibiting dilated congested red pulp and areas of hemorrhage (red arrows) with atrophic white pulp (black arrow). (**c**) The standard drug-treated group displayed no congestion in the red pulp (red arrow) with average-sized lymphoid follicles (black arrow). (**d**) Diclofenac sodium group (20 mg/kg) displayed moderate congestion in the red pulp (blue arrow) with hemorrhage (red arrow) and small-sized lymphoid follicles (black arrow). (**e**) Diclofenac sodium group (40 mg/kg) displayed mild congestion in the red pulp (blue arrow), a small area of hemorrhage (red arrow) and average-sized lymphoid follicles (black arrows). 100× magnification.

**Fig. 7:**
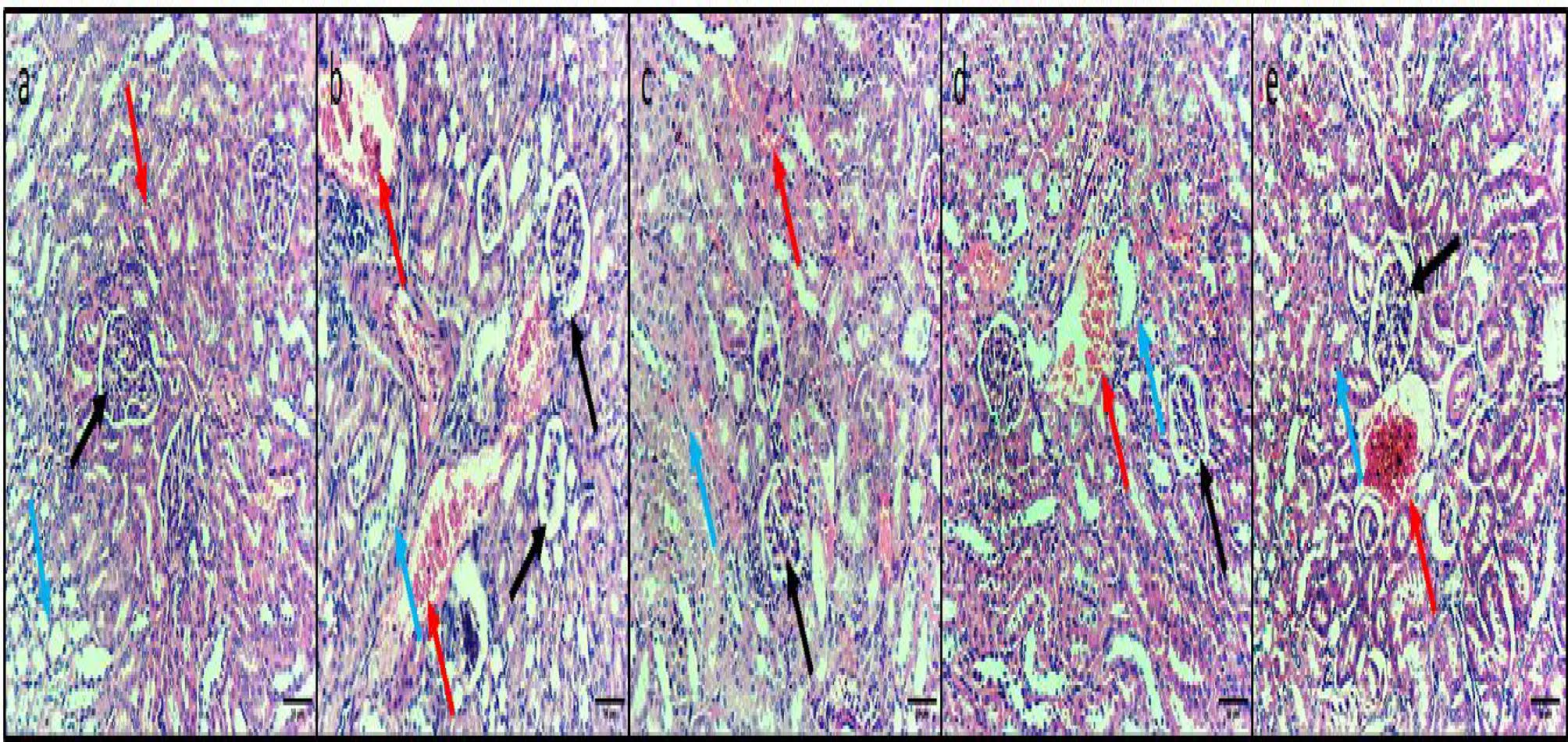
H&E micrographs of kidney sections of (**a**) Normal control group displaying glomerulus with usual appearance (black arrow), distal tubules (blue arrow) and proximal tubules (red arrow) with usual structure. (**b**) The positive control group displayed glomerulus shrinkage with widening of Bowman’s capsule (black arrow), marked congestion and hemorrhage (red arrow), and tubular necrosis (blue arrow). (**c**) The standard drug-treated group displayed glomeruli with the usual appearance (black arrow), normal dentinal tubules (blue arrow), and minimal interstitial congestion (red arrow). (**d**) Diclofenac sodium group (20 mg/kg) displayed glomerulus shrinkage with widening of Bowman’s capsule (black arrow), moderate congestion (red arrow), and tubular necrosis (blue arrow). (**e**) The diclofenac sodium group (40 mg/kg) displayed normal-sized glomeruli (black arrow), mild congestion (red arrow), and normal kidney tubules (blue arrow). 200× magnification.

Diclofenac sodium (40 mg/kg) was revealed to significantly improve the histological architecture of the liver, spleen, and kidney after triggering a systemic infection in mice, as displayed in Figs. 5 and 6, and 7.

### In Silico studies

The binding affinity and binding mechanisms for diclofenac sodium on three proteins associated with *S. epidermidis* were explored using molecular docking. The tested ligand exhibited variable binding affinities to the target proteins under investigation, as designated by the docking score (Table 3).

**Table 3:** Docking affinities (Kcal/mol) were used to study compounds in proteins of *S. epidermidis*.

Protein	S. epidermidis protein	IcaC	TcaR
Docking affinities	−7.2	−6.1	−7.1

**Fig. 8:**
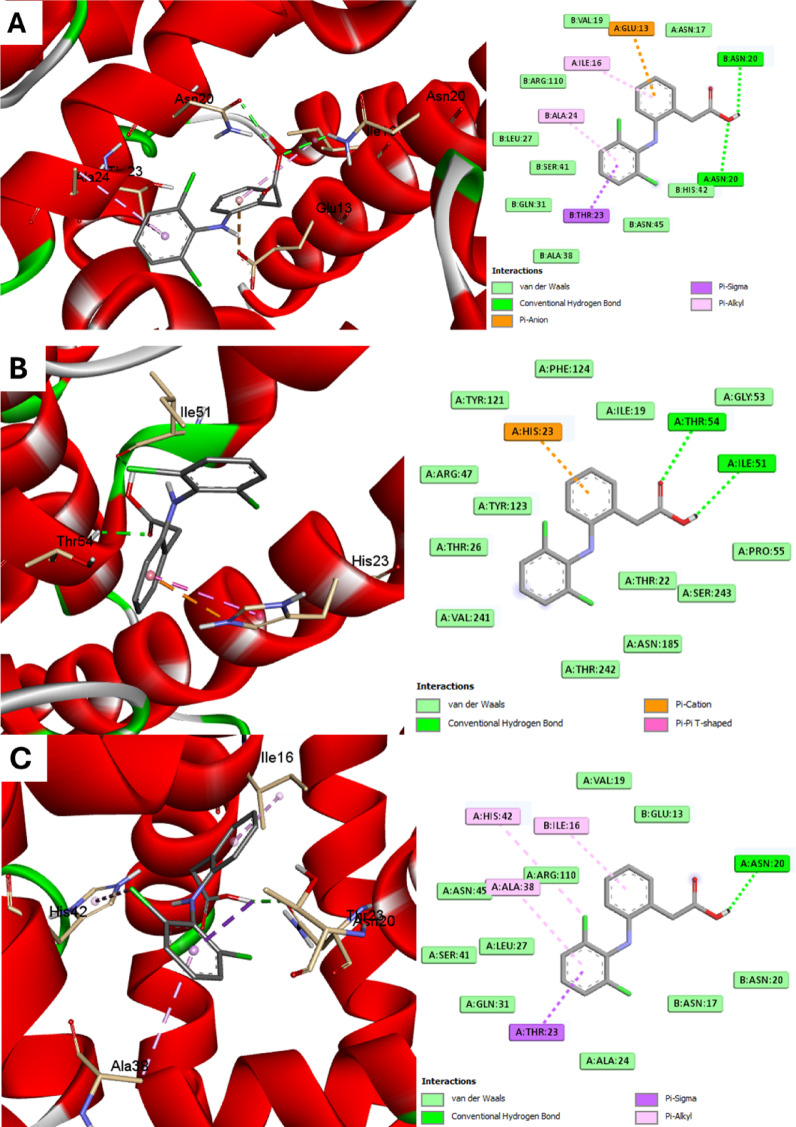
Docking of diclofenac sodium into proteins of *S. epidermidis* (**A**) *S. epidermidis* protein (code: 3KP3), (**B**) IcaC (AlfaFold: AF-A0A162GV30-F1) and (**C**) TcaR (code: 3KP4) enzymes. The figures were created by AutoDock Vina software and ligand structures were drawn into Marvin Sketch V22.2.

According to the docking affinities, diclofenac sodium presented a greater affinity to *S. epidermidis* protein and TcaR enzymes, while a lower affinity was associated with the IcaC enzyme. Diverse forces were involved: H-bonding, hydrophobic interaction, Pi-sigma, Pi-anion and Pi-cation interactions (Fig. 8).

After all, good affinities were obtained with the three enzymes of *S. epidermidis*, with *S. epidermidis* protein and TcaR being the highest. Diclofenac showed good affinities, suggesting the possible mechanisms for its action on *S. epidermidis*.

## Discussion

The capacity of bacterial cells to reveal resistance to antibiotics denotes a major global healthcare problem, which is augmented by the reduced supply of innovative antibiotics^46–48^. Antibiotic-resistant bacteria often trigger severe infections that increase hospitalization, healthcare expenses, morbidity, and mortality. Therefore, novel plans to stop such matters are mandatory^49^. Here, *S. epidermidis* obtained from different specimens revealed multidrug resistance to various antibiotics, which is comparable with the data reported in previous studies^50–52^.

Repurposing the FDA-approved drugs to manage the infections is a substitutional approach to hasten the development of innovative antimicrobials^53,54^. Recently, certain studies recognized the antimicrobial action of numerous non-antibiotic drugs^55^. Nonsteroidal anti-inflammatory drugs (NSAIDs) possess analgesic, anti-inflammatory, and antipyretic action. Interestingly, NSAIDs have been previously found to have antibacterial action toward different bacterial species^56^. In addition, their potential to impede biofilm formation was elucidated^57^. Diclofenac sodium is one of the NSAIDs investigated in the current study for its antibacterial and antibiofilm action on *S. epidermidis* clinical isolates. It has exposed antibacterial action on *S. epidermidis* isolates with MICs that ranged from 500 to 2000 µg/mL. Earlier studies described the antibacterial action of diclofenac sodium on *Enterococcus faecalis*^58^ and *S. aureus*^38,56^.

Biofilm formation is an important virulence factor that triggers antibiotic resistance^59,60^. *S. epidermidis* can form biofilms that may influence humans or be formed on medical devices, triggering persistent infections. This is attributed to the augmented antibiotic resistance that may be 1000 times that of the planktonic cells^12,61^. In this study, diclofenac sodium showed antibiofilm action by crystal violet assay, which was confirmed using SEM. A previous study reported the antibiofilm action of diclofenac sodium on *S. aureus* biofilm^56^ and polymicrobial endodontic biofilm^62^.

Bacteria exposed to diclofenac sodium at sub-MIC considerably declined the expression of *cna*, *fnb*A, and *ica* genes in 20% of the isolates. During the early steps of biofilm formation, bacteria often anchor themselves onto biomaterial surfaces via specific extracellular matrix proteins called adhesins. Fibronectin-binding protein A (FnbA), encoded by *fnb*A, and collagen-binding protein (Cna), encoded by *the cna* gene, are among such adhesins^63^. Also, the intercellular adhesion (*ica*) gene is essential in biofilm formation^64^. Thus, downregulating such genes would have a great value in the antibiofilm action of diclofenac sodium.

To confirm diclofenac sodium’s capability to fight the infections triggered by *S. epidermidis*, we evaluated the consequences of diclofenac sodium on the systemic infection in mice. Remarkably, diclofenac sodium diminished the liver’s infiltrating inflammatory cells and apoptotic bodies. Also, diclofenac sodium reduced the congestion and hemorrhage in the spleen and kidney tissues and restored the normal conditions of glomeruli and tubules in the kidney. Although the pathogenic bacteria trigger infection, inflammation is one of the host’s reactions to this pathogen. Thus, one therapeutic effect of the antibacterial agent is to decrease the inflammatory response^65^, which could be endorsed by the anti-inflammatory action of diclofenac sodium.

Regarding the molecular docking studies, good affinities were observed with the three possible targets of *S. epidermidis* and the *S. epidermidis* protein and TcaR being the highest, proposing the probable mechanisms for the action of diclofenac sodium on *S. epidermidis*. To our knowledge, this is the first time that the docking affinities of diclofenac sodium on the studied targets in *S. epidermidis* have been revealed.

## Conclusion

In this study, diclofenac sodium revealed antibacterial and antibiofilm action on the tested *S. epidermidis* isolates. The in vitro studies employed agar well diffusion, broth microdilution, SEM, and qRT-PCR. Regarding the in vivo studies, a systemic infection was triggered in mice to simulate the human body. In addition, in silico studies were performed. Thus, diclofenac sodium can suppress bacterial growth and its ability to form biofilm. It may serve as a drug candidate for antimicrobial and anti-biofilm agents that need further clinical studies soon.

## Data Availability

All data are available in the manuscript and supporting materials.

## References

[1] Singh, R., Ray, P., Das, A. & Sharma, M. Penetration of antibiotics through Staphylococcus aureus and Staphylococcus epidermidis biofilms. *J. Antimicrob. Chemother.* **65**, 1955–1958 (2010). 20615927 10.1093/jac/dkq257

[2] Amorena, B. et al. Antibiotic susceptibility assay for Staphylococcus aureus in biofilms developed in vitro. *J. Antimicrob. Chemother.* **44**, 43–55 (1999). 10459809 10.1093/jac/44.1.43

[3] Jarraud, S. et al. Relationships between Staphylococcus aureus genetic background, virulence factors, Agr groups (alleles), and human disease. *Infect. Immun.* **70**, 631–641 (2002). 11796592 10.1128/IAI.70.2.631-641.2002 PMC127674

[4] Males, B. M., Rogers, W. A. Jr & Parisi, J. T. Virulence factors of biotypes of Staphylococcus epidermidis from clinical sources. *J. Clin. Microbiol.* **1**, 256–261 (1975). 1176603 10.1128/jcm.1.3.256-261.1975 PMC275050

[5] Datta, S., Singh, V., Nag, S. & Roy, D. N. Carvacrol, a monoterpenoid, binds quorum sensing proteins (LasI and LasR) and swarming motility protein BswR of Pseudomonas aeruginosa, resulting in loss of pathogenicity: an in Silico approach. *Can. J. Microbiol.* **71**, 1–15 (2024). 10.1139/cjm-2024-0155 39566032

[6] Moktan, N. et al. Antibacterial and antibiofilm activities of extract and bioactive compounds from Bergenia ciliata (Haw.) sternb. Flowers against Streptococcus mutans through cell membrane damage. *J. Ethnopharmacol.* **339**, 119144 (2025). 39577678 10.1016/j.jep.2024.119144

[7] Roy, D. N. *A complete guidebook on biofilm study*; Academic Press: (2022).

[8] Branda, S. S., Vik, Å., Friedman, L. & Kolter, R. Biofilms: the matrix revisited. *Trends Microbiol.* **13**, 20–26 (2005). 15639628 10.1016/j.tim.2004.11.006

[9] Attallah, N. G. et al. Anti-Biofilm and antibacterial activities of Cycas media R. Br secondary metabolites: in silico, in vitro, and in vivo approaches. *Antibiotics* **11**, 993 (2022). 35892383 10.3390/antibiotics11080993 PMC9394325

[10] Datta, S., Singh, V., Nag, S. & Roy, D. N. Marine-Derived cytosine arabinoside (Ara-C) inhibits biofilm formation by inhibiting PEL Operon proteins (Pel A and Pel B) of Pseudomonas aeruginosa: an in Silico approach. *Molecular Biotechnology* **67**1–15. (2024). 10.1007/s12033-024-01169-8 38739212

[11] Livermore, D. M. Antibiotic resistance in Staphylococci. *Int. J. Antimicrob. Agents*. **16**, 3–10 (2000). 10.1016/s0924-8579(00)00299-5 11137402

[12] Mah, T. F. C. & O’Toole, G. A. Mechanisms of biofilm resistance to antimicrobial agents. *Trends Microbiol.* **9**, 34–39 (2001). 11166241 10.1016/s0966-842x(00)01913-2

[13] Hansa, R. K. et al. 4-4-(Anilinomethyl)-3-[4-(trifluoromethyl) phenyl]-1H-pyrazol-1-ylbenzoic acid derivatives as potent anti-gram-positive bacterial agents. *Eur. J. Med. Chem.* **219**, 113402 (2021). 33845234 10.1016/j.ejmech.2021.113402 PMC8165011

[14] Provenzani, R. et al. Multisubstituted pyrimidines effectively inhibit bacterial growth and biofilm formation of Staphylococcus aureus. *Sci. Rep.* **11**, 7931 (2021). 33846401 10.1038/s41598-021-86852-5 PMC8041844

[15] Al-Fakhrany, O. M. & Elekhnawy, E. Helicobacter pylori in the post-antibiotics era: from virulence factors to new drug targets and therapeutic agents. *Arch. Microbiol.* **205**, 301 (2023). 37550555 10.1007/s00203-023-03639-0 PMC10406680

[16] Payne, D. J., Gwynn, M. N., Holmes, D. J. & Pompliano, D. L. Drugs for bad bugs: confronting the challenges of antibacterial discovery. *Nat. Rev. Drug Discovery*. **6**, 29–40 (2007). 17159923 10.1038/nrd2201

[17] Ribeiro da Cunha, B., Fonseca, L. P. & Calado, C. R. Antibiotic discovery: where have we come from, where do we go? *Antibiotics* **8**, 45 (2019). 31022923 10.3390/antibiotics8020045 PMC6627412

[18] Talat, A., Bashir, Y. & Khan, A. U. Repurposing of antibiotics: sense or non-sense. *Front. Pharmacol.* **13**, 833005 (2022). 35264965 10.3389/fphar.2022.833005 PMC8900814

[19] Boyd, N. K., Teng, C. & Frei, C. R. Brief overview of approaches and challenges in new antibiotic development: a focus on drug repurposing. *Front. Cell. Infect. Microbiol.* **11**, 684515 (2021). 34079770 10.3389/fcimb.2021.684515 PMC8165386

[20] Forbes, B. A., Sahm, D. F. & Weissfeld, A. S. *Diagnostic microbiology*; Mosby St Louis: (2007).

[21] Al-Zoubi, M. S., Al-Tayyar, I. A., Hussein, E., Al Jabali, A. & Khudairat, S. Antimicrobial susceptibility pattern of Staphylococcus aureus isolated from clinical specimens in Northern area of Jordan. *Iran. J. Microbiol.* **7**, 265 (2015). 26719783 PMC4695508

[22] Guan, C. et al. Antibacterial and antibiofilm potential of Lacticaseibacillus rhamnosus YT and its cell-surface extract. *BMC Microbiol.* **23**, 1–10 (2023). 36635630 10.1186/s12866-022-02751-3 PMC9835366

[23] Alotaibi, B. et al. Aqueous core Epigallocatechin gallate PLGA nanocapsules: characterization, antibacterial activity against uropathogens, and in vivo reno-protective effect in cisplatin induced nephrotoxicity. *Drug Deliv.* **29**, 1848–1862 (2022). 35708451 10.1080/10717544.2022.2083725 PMC9225707

[24] Negm, W. A. et al. Promising antifungal activity of encephalartos Laurentianus de wild against Candida albicans clinical isolates: in vitro and in vivo effects on renal cortex of adult albino rats. *J. Fungi*. **8**, 426 (2022). 10.3390/jof8050426 PMC9144060 35628682

[25] Alqahtani, M. J., Elekhnawy, E., Negm, W. A., Mahgoub, S. & Hussein, I. A. Encephalartos villosus lem. Displays a strong in vivo and in vitro antifungal potential against Candida glabrata clinical isolates. *J. Fungi*. **8**, 521 (2022). 10.3390/jof8050521 PMC9146621 35628776

[26] David, V. et al. Validation of a method of broth microdilution for the determination of antibacterial activity of essential oils. *BMC Res. Notes*. **14**, 1–7 (2021). 34857039 10.1186/s13104-021-05838-8 PMC8638534

[27] Binsuwaidan, R. et al. Bilosomes as nanoplatform for oral delivery and modulated in vivo antimicrobial activity of lycopene. *Pharmaceuticals* **15**, 1043 (2022). 36145264 10.3390/ph15091043 PMC9505130

[28] Elekhnawy, E. A., Sonbol, F. I., Elbanna, T. E. & Abdelaziz, A. A. Evaluation of the impact of adaptation of Klebsiella pneumoniae clinical isolates to Benzalkonium chloride on biofilm formation. *Egypt. J. Med. Hum. Genet.* **22**, 1–6 (2021).38624675

[29] Stepanović, S. et al. Quantification of biofilm in microtiter plates: overview of testing conditions and practical recommendations for assessment of biofilm production by Staphylococci. *Apmis* **115**, 891–899 (2007). 17696944 10.1111/j.1600-0463.2007.apm_630.x

[30] Alherz, F. A. et al. Silver nanoparticles prepared using encephalartos Laurentianus de wild leaf extract have inhibitory activity against Candida albicans clinical isolates. *J. Fungi*. **8**, 1005 (2022). 10.3390/jof8101005 PMC9604723 36294570

[31] Abdel Bar, F. M. et al. Anti-quorum sensing and anti-biofilm activity of pelargonium× hortorum root extract against Pseudomonas aeruginosa: combinatorial effect of Catechin and Gallic acid. *Molecules* **27**, 7841 (2022). 36431942 10.3390/molecules27227841 PMC9695561

[32] Ong, T. H. et al. Cationic chitosan-propolis nanoparticles alter the zeta potential of S. epidermidis, inhibit biofilm formation by modulating gene expression and exhibit synergism with antibiotics. *PloS One*. **14**, e0213079 (2019). 30818374 10.1371/journal.pone.0213079 PMC6394969

[33] Nourbakhsh, F. & Namvar, A. E. Detection of genes involved in biofilm formation in Staphylococcus aureus isolates. *GMS Hygiene and infection control ***11****.** (2016). 10.3205/dgkh000267 PMC4804124 27303652

[34] Atshan, S. S. et al. Quantitative PCR analysis of genes expressed during biofilm development of methicillin resistant Staphylococcus aureus (MRSA). *Infect. Genet. Evol.* **18**, 106–112 (2013). 23669446 10.1016/j.meegid.2013.05.002

[35] Atshan, S. S. et al. Quantitative PCR analysis of genes expressed during biofilm development of methicillin resistant Staphylococcus aureus (MRSA). *infection, genetics, and evolution ***18**, 106–112. (2013). 10.1016/j.meegid.2013.05.002 23669446

[36] Nourbakhsh, F. & Namvar, A. E. Detection of genes involved in biofilm formation in Staphylococcus aureus isolates. *GMS Hygiene Infect. Control*. **11**, Doc07 (2016). 10.3205/dgkh000267 PMC4804124 27303652

[37] Zheng, J. et al. Differential gene expression by RamA in ciprofloxacin-resistant Salmonella typhimurium. *PLoS One*. **6**, e22161 (2011). 21811569 10.1371/journal.pone.0022161 PMC3139621

[38] Zhang, S. et al. Diclofenac resensitizes Methicillin-Resistant Staphylococcus aureus to β‐Lactams and prevents implant infections. *Adv. Sci.* **8**, 2100681 (2021). 10.1002/advs.202100681 PMC8261494 34258168

[39] Thompson, J. M. et al. Oral-only linezolid-rifampin is highly effective compared with other antibiotics for periprosthetic joint infection: study of a mouse model. *J. Bone Joint Surg. Am. Vol.* **99**, 656 (2017). 10.2106/JBJS.16.01002 PMC6181281 28419033

[40] Al-Kuraishy, H. M. et al. Potential therapeutic benefits of Metformin alone and in combination with sitagliptin in the management of type 2 diabetes patients with COVID-19. *Pharm* **15**, 1361 (2022). 10.3390/ph15111361 PMC9699540 36355535

[41] Trott, O. & Olson, A. J. AutoDock vina: improving the speed and accuracy of Docking with a new scoring function, efficient optimization, and multithreading. *J. Comput. Chem.* **31**, 455–461. 10.1002/jcc.21334 (2010). 19499576 10.1002/jcc.21334 PMC3041641

[42] Stackhouse, R. R., Faith, N. G., Kaspar, C. W., Czuprynski, C. J. & Wong, A. C. L. J. A. microbiology, e. Survival and virulence of Salmonella enterica serovar enteritidis filaments induced by reduced water activity. *Applied and environmental microbiology* **78**, 2213–2220 (2012). 10.1128/AEM.06774-11 PMC3302626 22287000

[43] Alomair, B. M. et al. Is sitagliptin effective for SARS-CoV-2 infection: false or true prophecy? *Inflammopharmacology* **30**, 2411–2415 (2022). 36180664 10.1007/s10787-022-01078-9 PMC9524728

[44] Chang, Y. M. et al. Structural study of TcaR and its complexes with multiple antibiotics from Staphylococcus epidermidis. *Proc. Natl. Acad. Sci. U.S.A.* **107**, 8617–8622. 10.1073/pnas.0913302107 (2010). 20421503 10.1073/pnas.0913302107 PMC2889327

[45] Hasan Khudhair, D. et al. Combination of vitamin C and Curcumin safeguards against methotrexate-induced acute liver injury in mice by synergistic antioxidant effects. *Front. Med.* **9**, 866343 (2022). 10.3389/fmed.2022.866343 PMC9047671 35492324

[46] Ventola, C. L. The antibiotic resistance crisis: part 1: causes and threats. *Pharm. Ther.* **40**, 277 (2015). PMC4378521 25859123

[47] Cegelski, L., Marshall, G. R., Eldridge, G. R. & Hultgren, S. J. The biology and future prospects of antivirulence therapies. *Nat. Rev. Microbiol.* **6**, 17–27 (2008). 18079741 10.1038/nrmicro1818 PMC2211378

[48] Defoirdt, T. Quorum-sensing systems as targets for antivirulence therapy. *Trends Microbiol.* **26**, 313–328 (2018). 29132819 10.1016/j.tim.2017.10.005

[49] Prestinaci, F., Pezzotti, P. & Pantosti, A. Antimicrobial resistance: a global multifaceted phenomenon. *Pathogens Global Health*. **109**, 309–318 (2015). 26343252 10.1179/2047773215Y.0000000030 PMC4768623

[50] Martínez-Santos, V. I., Torres-Añorve, D. A., Echániz-Aviles, G., Parra-Rojas, I. & Ramírez-Peralta, A. Castro-Alarcón, N. Characterization of Staphylococcus epidermidis clinical isolates from hospitalized patients with bloodstream infection obtained in two time periods. *PeerJ* **10**, e14030 (2022). 36213498 10.7717/peerj.14030 PMC9541613

[51] Ahmadunissah, A., Aazmi, S., Abd Hamid, U. M. & Abdul-Aziz, A. Multidrug resistance of Staphylococcus epidermidis: an emerging threat to global health. *J. Appl. Pharm. Sci.* **12**, 001–010 (2022).

[52] Siciliano, V., Passerotto, R. A., Chiuchiarelli, M., Leanza, G. M. & Ojetti, V. Difficult-to-Treat pathogens: A review on the management of Multidrug-Resistant Staphylococcus epidermidis. *Life* **13**, 1126 (2023). 37240771 10.3390/life13051126 PMC10220888

[53] Rangel-Vega, A., Bernstein, L. R., Mandujano-Tinoco, E. A., García-Contreras, S. J. & García-Contreras, R. Drug repurposing as an alternative for the treatment of recalcitrant bacterial infections. *Front. Microbiol.* **6**, 282 (2015). 25914685 10.3389/fmicb.2015.00282 PMC4391038

[54] Thangamani, S., Mohammad, H., Younis, W. & Seleem, N. Drug repurposing for the treatment of Staphylococcal infections. *Curr. Pharm. Design*. **21**, 2089–2100 (2015). 10.2174/1381612821666150310104416 PMC8672279 25760334

[55] Lagadinou, M. et al. Antimicrobial properties on non-antibiotic drugs in the era of increased bacterial resistance. *Antibiotics* **9**, 107 (2020). 32131427 10.3390/antibiotics9030107 PMC7175110

[56] Leão, C., Borges, A. & Simões, M. NSAIDs as a drug repurposing strategy for biofilm control. *Antibiotics* **9**, 591 (2020). 32927675 10.3390/antibiotics9090591 PMC7558876

[57] Barbarossa, A. et al. Non-antibiotic drug repositioning as an alternative antimicrobial approach. *Antibiotics* **11**, 816 (2022). 35740222 10.3390/antibiotics11060816 PMC9220406

[58] Salem-Milani, A. et al. Antibacterial effect of diclofenac sodium on Enterococcus faecalis. *J. Dentistry (Tehran Iran)*. **10**, 16 (2013). PMC3666061 23724199

[59] Foster, T. J. Antibiotic resistance in Staphylococcus aureus. Current status and future prospects. *FEMS Microbiol. Rev.* **41**, 430–449 (2017). 28419231 10.1093/femsre/fux007

[60] Petchiappan, A. & Chatterji, D. Antibiotic resistance: current perspectives. *ACS Omega*. **2**, 7400–7409 (2017). 30023551 10.1021/acsomega.7b01368 PMC6044581

[61] Otto, M. Staphylococcal biofilms. *Bacterial biofilms* , 207–228 2008. 10.1007/978-3-540-75418-3_10 PMC2777538 18453278

[62] Ferrer-Luque, C. M., Solana, C., Aguado, B. & Ruiz-Linares, M. Antimicrobial activity and cytotoxicity of nonsteroidal Anti-Inflammatory drugs against endodontic biofilms. *Antibiotics* **12**, 450 (2023). 36978317 10.3390/antibiotics12030450 PMC10044545

[63] Arciola, C. R., Campoccia, D., Gamberini, S., Baldassarri, L. & Montanaro, L. Prevalence of Cna FnbA and FnbB adhesin genes among Staphylococcus aureus isolates from orthopedic infections associated to different types of implant. *FEMS Microbiol. Lett.* **246**, 81–86 (2005). 15869965 10.1016/j.femsle.2005.03.035

[64] Nasr, R. A., AbuShady, H. M. & Hussein, H. S. Biofilm formation and presence of IcaAD gene in clinical isolates of Staphylococci. *Egypt. J. Med. Hum. Genet.* **13**, 269–274 (2012).

[65] Signore, A. About inflammation and infection. *EJNMMI Res.* **3**, 8 (2013). 23374699 10.1186/2191-219X-3-8 PMC3564704

